# Uptake of Measles Second Dose Vaccine and Its Associated Factors Among Children Age 24−35 Months in Merhabete Woreda, Ethiopia: 2022: A Cross‐Sectional Study

**DOI:** 10.1002/hsr2.70278

**Published:** 2024-12-17

**Authors:** Ababye Mulatu Wmeskel, Melese Wagaye Zergaw, Hailegiyorgis Geleta Abocherugn

**Affiliations:** ^1^ Department of Public Health College of Medicine and Health Sciences, Wollo University Dessie Ethiopia; ^2^ Department of Nursing Debre Berhan University Asrat Woldeyes Health Sciences Campus Debre Berhan Ethiopia; ^3^ Department of Midwifery Mizan Tepi University College of Health Sciences Tepi Ethiopia

**Keywords:** factors, measles, second dose

## Abstract

**Background and Aim:**

Measles infection in children causes a high degree of morbidity and mortality. Vaccination with two doses of measles vaccine is the best strategy to prevent infection and its spread to other children. However, measles containing vaccination coverage in Ethiopia is below the WHO elimination goal. In this study, our aim was to assess uptake of measles second dose and its associated factors among children's age 24−35 months in Merhabete, North Shoa, Ethiopia, 2022.

**Methods:**

A community‐based cross‐sectional study design was conducted in Merhabete Woreda from June 1 to 30, 2022. A total of 732 children aged 24−35 months were included using a multistage and systematic random sampling technique. Data were collected using a semi‐structured, interviewer‐administered questionnaire. Verbal informed consent was taken regarding their willingness to participate in the study from the guardians. The information was kept confidential and not shared with a third party. Data entered into EpiData version 4.6 and exported to SPSS version 25. A logistic regression model was used to identify associated factors. AOR with 95% CI and *p* < 0.05 were used to declare statistically significant variables.

**Results:**

A total of 732 children with a response rate of 94.6% were included; the median age was 28 months. Uptake of measles vaccine second dose was 63.3% (95% CI 60%−67%). Knowledge of measles second dose schedule (AOR = 2.151, 95% CI 1.053−4.396), birth at a healthcare facility (AOR = 3.502, 95% CI 1.58−7.77), > 4 antenatal care visits (ANC) (AOR = 2.56, 95% CI 1.254−5.225), having immunization card (AOR = 9.958 (4.256−23.298), and mother age 25−34 years (AOR = 2.954, 95% CI 1.298−6.721) were significantly associated with the uptake of measles second dose.

**Conclusion and Recommendation:**

The uptake of measles second dose vaccine in Merhabete Woreda was below the WHO measles elimination target of > 95%. Variables such as number of antenatal follow‐up visits, institutional delivery, knowing the schedule of measles second dose vaccine, maintaining immunization card records, and maternal age were found to be independent predictors of vaccination. Therefore, Woreda Health Office managers should strengthen periodic monitoring and evaluate the implementation of measles second dose vaccine. Health professionals should also improve mother's awareness on measles second dose vaccine schedule and its importance and retain their immunization chart booklet.

**Abbreviations**
AEFI: adverse events following immunization
BCG: Tuberculosis vaccine (Bacillus‐Calmette‐Guerin)
CDC: Centers for Disease Control and Prevention
DHS: Demographic and Health Survey
DPT: diphtheria−pertussis−tetanus vaccine
EFY: Ethiopian fiscal year
EHNRI: Ethiopian Health and Nutrition Research Institute
GAVI: Global Alliance for Vaccine and Immunization
GIVS: Global Immunization Vision and Strategies
MCV: measles containing vaccine
SEPI: Expanded Program on Immunization

## 1 Introduction

Measles is a highly contagious viral disease that primarily affects children [1]. It is caused by a virus belonging to the paramyxovirus family and is typically spread through direct contact and the air [2]. Dehydration from severe diarrhea, malnourishment, otitis media, pneumonia, encephalitis (infection of the brain), and even death are among the complications of measles disease [3].

Measles is prevented by immunization with measles‐containing vaccine (MCV) [1]. Two doses of the vaccine are recommended to ensure immunity and prevent outbreaks, as about 15% of vaccinated children fail to develop immunity from the first dose [2]. Before the measles vaccine being developed in 1963, there were significant epidemics roughly every 2−3 years. It is estimated that 30 million cases of measles and over 2 million deaths from the disease occur annually worldwide, and that by the time a person turns 15 years old, more than 95% of them will had contracted the virus [3]. Reduced measles fatalities have been largely attributed to increased immunization efforts. A measles vaccination is estimated to have prevented 23.2 million deaths between 2000 and 2018. Measles mortality worldwide has dropped from an estimated 536,000 in 2000 to 142,000 in 2018, a 73% decrease [4].

Measles is extremely contagious, thus to stop its spread, two doses of the vaccine given on time and a very high vaccination rate ( > 95%) are needed The most important preventive strategy for effectively eradicating measles worldwide is maintaining high immunization rates with two doses of the vaccine [5]. Among European nations, the average coverage rate for the measles second dose vaccine was 89.9% between 2015 and 2017. Of these, five countries—Croatia (95.7%), Portugal (95.0%), Hungary (99.0%), Slovakia (97.0%), and Sweden (95.0%)—had coverage rates for the vaccine that exceeded the measles elimination target of > 95% [5]. The coverage of the second dose of measles by WHO areas varies from 26% in the African region to 91% in the Western Pacific and European regions among all the nations that have incorporated MCV2 into their regular immunization schedule [6]. MCV2 coverage was estimated to be 71% worldwide in 2019; however, because of the COVID‐19 pandemic in 2020, this number dropped to 70%. Slovakia (97.0%), Sweden (95.0%), and Hungary (99.0%) [7].

As of December 2016, among countries in the African Region have WHO/UNICEF estimates of MCV2 coverage available in 2015, Algeria, Seychelles, and Cabo Verde have coverage of ≥ 95%, Botswana, Lesotho, Mauritius, Rwanda, and Swaziland have coverage of > 80%; while Niger 16%, Kenya 28%, Senegal 54%, and Zambia 47% [8]. As evidenced by the joint report of WHO and UNICEF, the second‐dose MCV coverage in Ethiopia was 71, and 70.56% in 2020 and 2021, respectively [9]. The overall second dose of measles vaccination use was 48.1% in a community‐based cross‐sectional survey carried out in Jabitehnan District, Northwest Ethiopia, between September 1, 2020, and October 1, 2020 [10]. In Northwest Ethiopia, a community‐based cross‐sectional research carried out in January and February of 2022 found that 41.39% of children aged 24−35 months had received the second dose of the measles vaccine [11].

Low coverage of MCV2 in the second year of life increases the accumulation of susceptible children who did not respond to MCV1 or did not receive the first dose, this leads to resurgence of measles outbreak; hence it cause increased childhood morbidity and mortality with measles diseases and its complication like pneumonia, diarrhea, and malnutrition [4].

Research findings indicate that the combined use of MCV1 and MCV2 two doses is significantly more effective in avoiding the disease than using only one dose. It is advised that all nations incorporate routine MCV2 immunization into their national immunization schedules, irrespective of the extent of coverage with a normal dose of MCV1. By expanding the distribution of the measles vaccination to include two regular doses, numerous nations have effectively eradicated the virus. However, a variety of factors have prevented measles from being completely eradicated, and it still ranks high among the causes of young deaths in developing nations [12].

Different factors determine the uptake of measles's second dose. A study in China reported that maternal low educational level, fixed job, and place of delivery significantly affected measles second dose [13]. According to a study in Kenya, distance to immunization delivery site, knowledge of measles second dose schedule, and coverage of other vaccine (penta_3_.measles first dose) were factors for uptake of MCV second dose [14, 15].

From 2000 to 2022, the measles immunization prevented 57 million fatalities. Despite the availability of a safe and affordable vaccine, a projected 136,000 measles deaths occurred worldwide in 2022, with children under the age 5 that were either unvaccinated or under vaccinated. In 2023, 83% of children received their first dose of the measles vaccine, a significant decrease from the 86% level in 2019 [16].

The immunization agenda 2020−2030 set by WHO robust measles surveillance systems to document immunity gaps, identify root causes of under vaccination, and develop locally tailored solutions to ensure administration of second doses of MCV to all children [17]. Despite the significant reduction in measles mortality, the reality is that measles vaccination coverage, the quality of measles supplementary immunization activities, and the quality of disease surveillance in the African Region have not yet reached the levels required to avert the resurgence of measles [18].

Although a number of studies have reported findings on full vaccination with BCG, OPV, Penta, and MCV1 among the age of 12−23 months, none of them generate evidence about the uptake and factors that influence the uptake of MCV2 separately in Ethiopia. The lack of information could have been attributed to the fact that MCV2 was introduced into routine immunization (RI) program just a few years ago in country. In Ethiopia, MCV2 was introduced in February 2019 [19]. Consequently, there is limited information on the uptake of measles second dose across the country and including the study area. This study is aimed to determine and identify factors associated with the uptake of measles second dose among children aged 24−35 completed month, in Merhabete Woreda, North Shoa Zone, Amhara Region, Ethiopia, 2022.

## 2 Methods and Materials

### 2.1 Study Area

The study was conducted in Merhabete Woreda, North Shoa zone of Amhara regional state. It is found 735 km from Bahir Dar and 183 km from Addis Ababa. The Woreda covers an area of approximately 5872.4 km^2^. It has 23 rural and 4 urban “kebeles” the lowest administrative unit, the projected population from 2013 to 2014 is 36,229 urban and 117,797 rural, total 154,026 with estimated number of surviving infant at 1 year 4790 and estimated children less than 3 years 12,037. The health service coverage of the Woreda is 1 general hospital, 6 health centers, and 23 rural health posts are currently provide basic health service for the catchment population. Immunization service has been administered through routine healthcare deliveries in governmental health facilities (hospitals, health centers, and health posts) and outreach at community level free of charge.

### 2.2 Study Design and Period

A community‐based quantitative cross‐sectional study was conducted from June 1 to 30, 2022.

### 2.3 Study Participants

All children aged 24−35 completed months of mothers or caregivers who were living in selected kebeles of Merhabete Woreda, North Shoa Zone, Ethiopia, respectively were included.

### 2.4 Sample Size Determination and Sampling Technique

The sample size was calculated using a single population proportion formula, *n* = Z$\propto \frac{1}{2}2\times P(1-P)/d2$. Since there were limited studies on the uptake of the second dose of measles vaccine in the country; considering 50% proportion, a 95% confidence level with 5% margin of error, design effect (DEFF), and nonresponse rate of 10%.

$$\text{Thus},\;Z^{2}p\left(1-p\right)/d^{2}=1.96\times 1.96\times 0.5\left(1-0.0.5\right)/0.05^{2}=384$$

The total number of children in the Woreda obtained from children growth monitoring and deworming register is 4072 which is less than 10,000. Using the population correction formula, nf=ni/1+(ni/N)= 352.3−352. Then 352 × DEFF, 352 × 2 = 704, adding 10% nonresponse rate 704 × 0.1 = 70.4; the required sample for the first objective was be 704 + 70 = 774.

A multistage sampling method was employed to select the study participants. Initially, the Woreda stratified into urban and rural kebele. Using the assumption of rule of thumb, 30% of kebeles were included in the study for two strata. In stages one and eight, kebele were selected by using a simple random sampling method (one from urban and seven from rural). List of selected kebele in order of selection from rural kebeles were Geren (180), Sergina (65), Delemma (223), Remeshit (235), Kolash (180), Buyo (168), Worego (218), and Alem Ketema 02 (377) from urban and total of 1646 children age 24−35 months obtained from child deworming registration book of the respective kebeles of the health post which was updated in March, 2014 EFY. Proportional sample size determination was used to allocate for each kebele. In stage two, HH with target children aged 24–35 months were selected by using a systematic random sampling technique. Then sampling interval *K* value was obtained. The first child in each kebele was selected randomly from the border of kebele, then rest of them were selected subsequently according to inclusion criteria based on the *K* interval until the required sample obtained. Women's Health Development Army (WHDA) was involved to guide households with eligible children in the respective kebele. For households with more than one child, the immunization status of the youngest child was assessed. If respondents were not found at home during the data collection, interviewers revisited the households for the second time and when the interviewers failed to find the eligible participant after two visits, declared as nonresponse.

### 2.5 Study Variables

The dependent variable was uptake of measles‐containing second dose vaccine, and the independent variables were sociodemographic‐related variables: Residence, maternal age, marital status, educational status, occupational status, age of the child, sex of child birth order, number of children, the decision to immunize the child in the HH, and relationship to the child. Behavioral‐related variables: ANC follow‐up, place of delivery, post‐natal visit, knowledge of measles second dose scheduled, knowledge, and attitude on vaccination. Health system‐related variables: Place of immunization, distance from immunization delivery site, adverse events following immunization, and utilization of other vaccines (Penta3, MCV1).

### 2.6 Operational Definitions

Uptake measles second dose (MCV2): A child who receives measles first dose (MCV1) at the age of 9−11 months and receives measles second dose at the age 15−23 months in RI service before the data collection period [1].

Good knowledge: Mother/caregiver/have above 75% of the knowledge score [20].

Moderate knowledge: Mother/caregiver/have 50%−75% of the knowledge score [20].

Poor knowledge: Mother/caregiver/have below 50% of total knowledge score [20].

Good attitude: Mother/caregiver/have above 50% of total attitude score [20].

Distance to immunization site: The time required to reach the immunization site on foot walk mother's judgment (perception).

Decision to immunization the child in the family: It was voluntary for the family to immunize the child as per the schedule mother/respondent judgment.

### 2.7 Data Collection Tools and Procedure

Two public health officers (PHO) working in EPI clinic of the health centers as supervisors and eight diploma health professionals as data collectors were recruited and training was given on objectives of the study, sampling technique and procedure, techniques of interview, data collection tools, and ethical issues. The data were collected through face‐to‐face interviews with the mothers/caregivers using a semi‐structured questionnaire adapted from min EDHS 2019 immunization questionnaire [21], a thorough review of literature [14, 15, 22], and through observation of the vaccination cards.

The questionnaire had three parts, sociodemographic related factor had 11 items, behavior related factors had 19 items (healthcare utilization of mother/care give/knowledge and attitude of mother/caregiver/on childhood vaccination and measles second dose vaccine related), and the health system related factors had 16 items. The knowledge and attitude assessment tool were adapted from [20]. The knowledge part initially had 12 items and it was reduced to nine due to three questions were not related to the objective of the study. The knowledge and attitude of the mother/caregiver/was assessed with two possible responses marked 1 and 0, where the response expressed having good knowledge and positive attitude. Regarding the immunization history of child, data was collected from immunization card or maternal recall; if child immunization card was available, data was recorded on the questionnaire. Mothers or caregivers who have no vaccination card for the child or if a vaccine had not been recorded on the card as being given, the mothers/caregivers were asked to recall the specific vaccines given to their child by reminding here type of vaccine, route of administration (injection, oral), number of dose given, and at what months of the child's age vaccines were administered. The questionnaire also contains outcome variable vaccinated for measles second dose coded as “1” and not vaccinated for second dose as “0.”

### 2.8 Data Quality Assurance

To ensure the validity of the data collection tool, the questionnaire was first prepared in English, then translated into the local language (Amharic) and finally back to English. Training was given to data collectors and supervisors by principal investigator on the overall data collection procedures and the techniques of interviewing for 1 day, a week before the actual data collection begins. The reliability of knowledge and attitude part of the data collection tool, was tested by Cronbach's *α* by the initial researcher, it was 0.89. To maintain the tool consistency, feasibility, and response rate, a pretest was conducted before 10 days of the actual data collection period among 40 (5%) in Agerite kebele of the Woreda which was not including in the final study; the questionnaire was corrected and some edition were made as per the result found. The questionnaire was given for EPI experts to assess content validity and give their expert input. For completeness, consistency, accuracy, and clarity, the collected data was checked by supervisors and the principal investigator daily.

### 2.9 Methods of Data Processing and Analysis

Data obtained from the questionnaire were cleaned, coded, and entered into Epi data software version 4.6, then exported to SPSS version 25 for analysis. Data were analyzed using bivariable and multivariable logistic regression at a 95% confidence interval (CI). After bivariable analysis, variable with *p* ≤ 0.25 was transferred to multivariable logistic regression and variable with a *p* < 0.05 was declared as statistically significant. Descriptive statistics were used, and the data were organized, processed, interpreted, and presented through words, tables, and graphs. The Hosmer–Lemeshow test was used to test goodness‐of‐fit.

## 3 Results

### 3.1 Sociodemographic Characteristics of Participant

A total of 732 children were evaluated with a response rate of 94.6%. Among them, 390 (53.3%) were male and the median age was 28 months with an interquartile range (IQR) of 26−32. Mothers/caregiver's as primary respondents participated in this study 509 (69.5%) were aged less than 35 years with median age of 30. Three hundred and ninety‐six (54.1%) were unable to read and write, 657 (89.8%) were married, and mothers as a caregiver contributed to higher proportion of 712 (97.3%) (Table 1).

**Table 1:** Childers's and mother's caregiver's sociodemographic characteristics in Merhabete Woreda, North Shoa Zone, Amhara Region, Ethiopia, 2022 (*n* = 732).

Variables	Response	Frequency	Percentage (%)
Sex of child	Male	390	53.3
	Female	342	46.7
Age of child	24−29 months	418	57.1
	30−35 months	314	42.9
Birth order	< 4	619	84.6
	4 and above	113	15.4
Age of the mother in years	15−24	127	17.3
	25−34	392	53.6
	More than 35	213	29.1
Residence	Urban	164	22.4
	Rural	568	77.6
Educational level of mother	Unable to read & write	396	54.1
	Primary (1−8)	211	28.8
	Secondary (9−12)	34	4.6
	Colleague & university	91	12.4
Marital status	Single	36	4.9
	Married	657	89.8
	Others*	39	5.3
Occupation	Housewife	120	16.4
	Farmer	457	62.4
	Other*	155	21.2
Relationship	Mother	712	97.3
	Grandmother	20	2.7
Decision to immunize the child	Both mother & father	557	76.1
	Others*	175	23.9
Number of children	Less than 4	610	83.3
	4 and above	122	16.7

* Marital status: widowed and divorced. Occupation: merchant and self‐employed, decision to immunize, mother only, and health professional.

### 3.2 Behavior Related Factors of Mother/Caregivers

**Figure 1:**
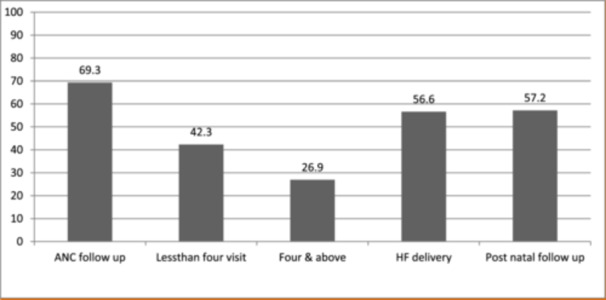
Behavioral‐related factors of MCV2 uptake among children aged 24−35 months in Merhabete Woreda North Shoa Zone, Amhara Region, Ethiopia, 2022.

Among the total participants, 508 (69.4%) had at least one antenatal follow‐up during the last pregnancy, of them; 414 (56.6%) were delivered in a health facility and 313 (42.8%) had PNC within the fourth five days of delivery (Figure 1).

### 3.3 Behavior Related Factors

**Figure 2:**
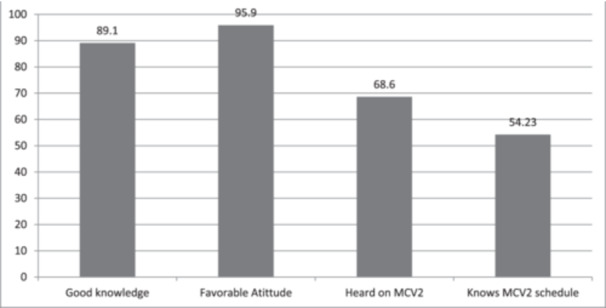
Behavioral‐related factors of MCV2 uptake among children aged 24−35 months in Merhabete Woreda, North Shoa Zone, Amhara Region, Ethiopia, 2022.

Mothers as a primary respondents 652 (89.1%) had good knowledge toward vaccination, 702 (95.9%) had a favorable attitude toward vaccination, 502 (68.0%) heard about the introduction of MCV second dose in RI, and 397 (54.2%) knew the schedule of MCV2 (Figure 2).

### 3.4 Health System Related Factors

Among 732 respondents, 445 (60.8%) vaccinated their child at outreach site, 576 (76.7%) immunization site within less than 30 min on foot walk, and 282 (38.5%) of the child experienced adverse events following immunization after receiving any dose of routine vaccination. The corresponding vaccine coverage of the children, 589 (80.5%) had vaccination chart booklet, 685 (93.6%) were vaccinated for BCG, 711 (97.1%) received OPV1, 649 (88.7%) received OPV3, 702 (95.9%) vaccinated for Penta1, 649 (88.7%) vaccinated for Penta3, 652 (89.1%) vaccinated for MCV1, and MCV2 463 (63.3%), respectively (Table 2).

**Table 2:** Health system‐related factors MCV2 uptake in Merhabete Woreda, North Shoa Zone, Amhara Region, Ethiopia, 2022 (*n* = 732).

Characteristics	Category	Frequency (*n*)	Percent (%)
Have immunization card/document	Yes	589	80.5
	No	143	19.5
	Outreach	445	80.4
	Health post	62	8.5
Place of immunization site	Health center	142	19.4
	Hospital	83	11.3
Distance of immunization site	Less than 30 min	576	76.7
	More than 30 min	156	21.3
Children develop AEFI after immunization	Yes	282	38.5
	No	450	61.5

### 3.5 Vaccination Status of Study Participants

**Figure 3:**
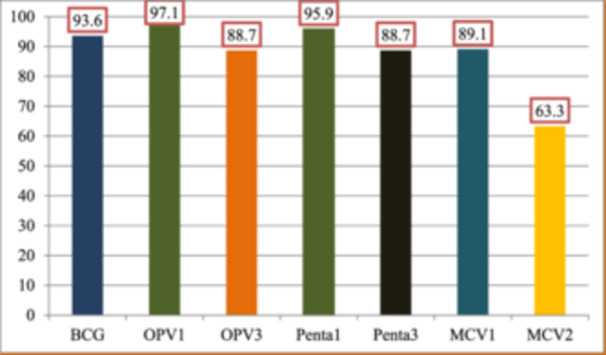
Vaccination status of children aged 24−35 months in Merhabete Woreda, North Shoa, Ethiopia, 2022.

Among 732 respondents, 63.3% of study participants were vaccinated for MCV2 second dose (Figure 3).

### 3.6 Reasons for Not Being Vaccinated for MCV2

Among 269 mothers/caregivers who did not vaccinate their children for MCV2, 178 (66.2%) responded due to lack of awareness on the necessity to return for second dose of MCV, 51 (18.96%) healthcare worker conceal (interrupt) immunization session, 30 (11.2%) lack of enough children to open the vial during immunization day, and 12 (4.5%) mothers/caregivers too busy (forget) were some reasons for not administering MCV2 in the Woreda.

### 3.7 Factors Associated With Uptake of MCV2

Bivariable logistic regression analysis was performed to identify variables associated with uptake of MCV2 at *p* < 0.25, and variables with *p* < 0.25 were enrolled in multivariable logistic regression. During bivariable logistic regression analysis, 14 variables were associated with the uptake of MCV2; those variables were residence, educational status, occupation, decision to vaccinate the child, ANC visit, place of delivery, PNC visit, knowledge of MCV2 schedule, availability of vaccination card, place of immunization site, distance to immunization site, number of children, and number of ANC visit, and they were enrolled to multivariable logistic regression. After adjustment for potential confounders, in multivariable analysis, mothers/caregivers knowledge of MCV2 schedule, health facility delivery, four and above ANC visits, availability of immunization card, and age of mother/caregiver 25−34 years were significantly associated with the uptake of MCV2 at *p* < 0.05.

The odds of MCV second dose uptake among children of mothers/caregivers, who knew the schedule of MCV2 dose was two times (AOR = 2.151, 95% CI: 1.053−4.396) more likely to receive MCV2 as compared to mothers/caregivers who did not know the schedule. Similarly, 77.05% (AOR = 3.502, 95% CI: 1.579−7.770), mothers who gave birth in the health facility were more likely to have taken MCV2 than mothers who delivered at home; the odds of MCV2 uptake in mothers/caregivers who had retained their immunization card was nine times (AOR = 9.958, 95% CI: 4.256−23.298) as compared to mothers/caregivers did not have a card or lost their card. Children whose mothers/caregiver were aged 25−34 years, were 2.9 times (AOR = 2.954, 95% CI: 1.298−6.721) more likely to vaccinate their child for MCV2. Moreover, children whose mothers had more than four ANC visits were 2.5 times (AOR = 2.56, 95% CI: 1.254−5.226) more likely to receiving MCV2, as compared to children whose mothers had not ANC follow‐up or less than four ANC visits; with the corresponding *p*‐value (Table 3).

**Table 3:** Bivariable and multivariable analysis of factors associated with MCV2 uptake in Merhabete Woreda, North Shoa, Zone, Ethiopia, 2022 (*N* = 732).

Variables	Categories	MCV2 vaccinated		COR (95% CI)	AOR (95% CI)	*p* value
		Yes	No			
Residence	Urban	128	36	2.473 (1.648−3.711)	1.810 (0.623−5.261)	0.276
	Rural	335	233	1.00		
Place of delivery	Home	144	174	1.00		
	HF	319	95	4.057 (2.952−5.577)	3.502 (1.579−7.77)	0.001*
PNC	Yes	239	74	2.812 (2.034−3.887)	1.373 (0.715−2.638)	0.341
	No	224	195	1.00		
MCV2 schedule knowledge	Yes	337	60	2.353 (1.425−3.884)	2.151 (1.053−4.396)	0.01*
	No	74	31	1.00		
Immunization card	Yes	426	163	7.487 (4942−11.344)	9.958 (4.256−23.298)	0.01
	No	37	106	1.00		
Place of immunization provided	Outreach	240	205	1.00		
	Health post	47	15	2.676 (1.454−4.928)	1.945 (0.631−6.00)	0.247
	Health center	107	35	2.611 (1.708−3.993)	0.844 (0.266−2.676)	0.773
	Hospital	69	14	4.21 (2.301−7.701)	0.292 (0.071−194)	0.087
Age of the mother	15−24 years	66	61	1.00		
	25−34 years	265	127	1.929 (1.283−2.898)	2.954 (1.298−6.721)	0.01*
	≥ 35 years	132	81	1.506 (0.966−2.35)	2.212 (0.826−5.924)	0.114
No of ANC follow‐up visit	≤ 3 visit	198	112	1.00		
	≥ visit	165	32	2.917 (1.871−4.546)	2.56 (1.254−5.226)	0.01*
Number of children	< 4	393	217	1.345 (0.906−1.998)	0.596 (0.202−1.76	0.349
	≥ 4	70	52	1.00		

* *p* < 0.01.

Abbreviations: AOR = adjusted odd ratio, CI = confidence interval, COR = crude odd ratio, and 1.00 as reference.

## 4 Discussion

The main preventive method for effective eradicating measles illness is sustained high vaccination coverage ( > 95%) with two timely doses of MCV [3]. When administered at the correct age and time interval, MCV are more effective [4]. WHO advises nations that aim to eradicate measles should achieve ≥ 95% coverage of all children in all areas with both doses (i.e., the first and the second dose) of all children in each Worwda [8].

The study found that 63.3% of children received the second dose of MCV with a 95% CI (60%−67%); this suggests that the remaining 36.7% of children had not received MCV2, which increases the number of unvaccinated children who are susceptible to the measles and may contribute to the outbreak of measles diseases. The result exceeded those of studies conducted in Pakistan (46.6%), China (44.7%), Kakamega Kenya (17.9%) [14] and Cherangany Sub County, Trans Nzoia County (56.6%) [15], and Tanzania 55.5% [23]. This gap may be explained by research participant characteristics, study period, sample size, study design, and accessibility of immunization services.

However, the uptake of MCV2 in this finding fell short of the > 95% coverage target for global elimination of measles [24]; a study in China, 93.9% [13], Sudan, 88% [22] and consistent with study in Indonesia 67% [25]. This could be as a result of variation in study participant characteristics, study period, sample size, accessibility to immunization service, and the recommendation of wastage rate for opening MCV across countries.

The coverage of MCV1 in this study was 89.1%; this shows that 25.8% of children failed to receive their second dose of MCV. The significant dropout rate from MCV1 and MCV2 may be due to the long duration between the first dose and the second dose which is wide apart; thus, the mother/caregiver is likely to forget the appointment or unaware of the schedule of MCV2; this suggests that poor communication between healthcare staff and the community about the appropriate ages for vaccination. This low coverage also shows that the Worwda is still at risk of measles outbreaks because the > 89.1% MCV1 coverage alone does not confer population immunity. Therefore, there is a need for MCV supplemental vaccination activities such as periodic intensification of routine immunization (PIRI) to improve population immunity. The MCV2 uptake can also be improved by establishing or strengthening the defaulter tracing system. Children who receive the first dose but fail to return for the second dose as scheduled are followed up and referred for vaccination.

The age of the mother (25−34 years) was significantly associated with the uptake of MCV2. It is supported by study in China, children whose mothers are younger has less chance to receiving the vaccine [13]. It is well recognized that age plays an important role in women's health services utilization, and maternal age may sometimes serve as a proxy for added knowledge of healthcare services. Age may be a factor which has a positive influence on accepting the full course of immunization programs. Moreover, mothers in this age group may have more knowledge and experience about healthcare services and value the health of their child, hence; may have good adherence to the full course of immunization than younger mothers.

Four and above ANC follow‐up during the pregnancy of the index child was significantly associated with the uptake of MCV2. If mother went to the health facility during pregnancy, health professionals would give advice and information on the progression of pregnancy, well‐being of baby, where should she deliver her baby and follow‐up care given for baby after birth. Mothers are also counseled on the importance of vaccination and when, where, and who gives the vaccine to her child immunization.

Health facility delivery was significantly associated with the uptake of MCV2. This finding was similar with the study in China and Malawi [13, 26]. This may be that mothers who gave birth at health institutions are closer to the health service provided. Health services packed provided after delivery includes maternal related health problems, family planning, and child health such as counseling on immunization programs, well baby clinic visits like growth monitoring; this may increase the mother's knowledge toward vaccination‐related issues, consequently health‐seeking behaviors of the mother improve and increase adherence to health service utilization including immunization program.

Availability of immunization card (booklet) or other document for vaccine provided was significantly associated with MCV2 uptake. This may be because availability of home‐based records may help the mother/caregiver to remember vaccination appointments; moreover, mothers/caregivers are more likely to make informed decisions.

Knowledge of the schedule of MCV2 among mothers/caregivers was significantly associated with the uptake of MCV2. This finding was supported by the study in Indonesia, Tanzania, Malawi, and Kenya [14, 15, 25, 26, 27]. This suggests mothers/caregivers having a better understanding of childhood vaccination schedules and reasons for vaccination may bring their child to vaccinate the subsequent dose more likely at the recommended time.

In contrast to the findings that have been documented in many studies [13, 14, 15, 22, 23, 28], residence, maternal educational level, occupation, and distance to immunization delivery site were not associated with the uptake of MCV2 dose in this study; this may be immunization service was equally accessible for all population by means of health extension program in the Worwda.

## 5 Conclusion and Recommendation

The uptake of MCV2 in Merhabete Worwda was below the measles elimination target > 95% and there was a significant dropout between MCV1 and MCV2. There was a likelihood of frequent measles cases in the County, thereby lowering the predictions for the vision of measles elimination unless a lot of emphasis is put on RI. Four and above ANC follow‐up visits, health facility delivery, knowledge of the schedule of MCV2, immunization card retention, and maternal age 25−34 years were found to be independent predictors for uptake of MCV2 in the Woreda. Therefore PIRI and supplemental immunization activity (SIAs), effective implementation, coordination and monitoring of community mobilization, awareness creation on routine vaccination, strengthening periodic Rapid Convenience Survey (RCS) at the household level, and implementation and monitoring community mobilization activities at the house level by means of WHDA is important to improve the uptake of MCV2.

## 6 Limitation

This study included the report on reviewing vaccination card and verbal report by the mother/Caregiver, which have significant difference in the uptake of MCV second dose between the two reports, which may have introduced recall bias. Data on the vaccination status and associated factors were collected at the same time; thus, it might not be possible to explain whether the factors preceded/influenced the vaccination status.

## Author Contributions

The ideal conception, study design, data acquisition, analysis, and interpretation were all contributed to by all of the authors. All of the authors have reviewed the manuscript and approved its publication in this journal.

## Ethics Statement

Wollo University Research and Ethical Review Committee approved the research proposal with a letter. Before data was collected, permission letters were obtained from the Woreda health office. Verbal informed consent has been taken from the guardian regarding their willingness to participate in the study. Participants' information was kept confidential and not shared with a third party. Participants that found to be sick or unvaccinated were referred to hospital or health center for treatment and vaccination. Above all, this study was entirely conducted as per the Declaration of Helsinki ethical principles for medical research on human participants.

## Consent

Verbal informed consent has been taken from the guardian regarding their willingness to participate in the study.

## Conflicts of Interest

The authors declare no conflicts of interest.

## Transparency Statement

The lead author, Melese Wagaye Zergaw, affirms that this manuscript is an honest, accurate, and transparent account of the study being reported, that no important aspects of the study have been omitted, and that any discrepancies from the study as planned (and, if relevant, registered) have been explained.

## Data Availability

The data supporting this study are made available within the manuscript.
